# Ultrafine Particle Recovery Using Thin Permeable Films

**DOI:** 10.3389/fchem.2018.00220

**Published:** 2018-06-19

**Authors:** Daniel J. Borrow, Kim van Netten, Kevin P. Galvin

**Affiliations:** Centre for Advanced Particle Processing, Newcastle Institute for Energy and Resources, University of Newcastle, Callaghan, NSW, Australia

**Keywords:** emulsion, hydrophobic, stabilization, permeable film, agglomeration, ultrafine particles

## Abstract

The selective recovery of ultrafine, <10 μm, particles remains a significant challenge in the minerals industry. Indeed, these particles often report to tailings impoundments, resulting in under-utilization of mined resources and the need for tailings dams. Recently, a technique has been developed offering the potential to selectively recover particles down to <1 μm in size. This technique, originally inspired by oil agglomeration, uses a high internal-phase water in oil emulsion as a binder to selectively agglomerate hydrophobic particles. Due to the significant concentration of the dispersed aqueous phase, ~95%, the continuous organic phase forms a network of very thin, permeable films, estimated to be 60 nm thick. These are stabilized by an emulsifier. In the high shear field of the agglomeration process, the binder is fragmented into smaller hydrophobic portions, delivering its thin film coating to the adhering hydrophobic particles. Permeation of water across the thin films eliminates the viscous hydrodynamic resistance, permitting sub-micron particle recovery to occur at rates similar to those for particles considerably larger in size. This recovery occurs within seconds under intense mixing. In this study, a model system consisting of magnetite, with a Sauter mean diameter of 11.4 μm, was agglomerated using the water in oil emulsion binder. The binder, which contained the emulsifier sorbitan monooleate, appeared to also act as a collector for the magnetite, thus no separate particle conditioning step was required. Curiously, however, the binder requirements were higher than expected. Further investigations concerning the stability of the binder revealed that the magnetite particles were causing rapid binder degradation. Therefore, prior to agglomeration using the binder, the particles were conditioned with sorbitan monooleate to render them hydrophobic. This pre-conditioning led to significant reductions in the binder dosage required to achieve agglomeration. Moreover, the resulting dosage matched that predicted by a model silica system for the same specific hydrophobic surface area, thus allowing a model to be validated based on the required binder dosage for a known hydrophobic surface area. Examination of binder stability in the presence of conditioned magnetite revealed that the now hydrophobic particles stabilized the binder.

## Introduction

Froth flotation has been widely used in the selective recovery of fine hydrophobic particles for around 100 years. The process allows large scale and economic processing of crushed minerals and ores to produce many of the metals needed in the modern world. Challenges are emerging however due to falling ore grades, and increased demand for base and precious metals. The flotation of ultrafine, <10 μm, particles has been shown to be inefficient and beyond the hydrodynamic limits of flotation (Leja, 2012), resulting in these particles reporting to tailings impoundments, representing a loss in valuable resources, and an environmental legacy associated with toxic heavy metals. Economies of scale have led to the need for increasingly larger process systems up to 650 m^3^ in volume (Wills and Finch, 2015). An alternative method for recovering and concentrating fine (<100 μm) and ultrafine (<1 μm) hydrophobic particles is oil agglomeration. The process, also called selective agglomeration, was originally developed for recovering fine coal from tailings, but has also been applied to tin, iron, and gold ores (Farnand et al., 1964, 1969; Sparks and Sirianni, 1974). Here, dispersed drops of oil are introduced, providing the hydrophobic interfaces in the same way air bubbles are used in flotation. Oil agglomeration offered many advantages, with higher recovery, improved kinetics, and the potential for dewatering using a mechanical screen (Mehrotra and Sastry, 1983). However, the process was never adopted across the industry due to the considerable cost of the oil (Miller, 1969). In an effort to economically recover ultrafine material, variations to both traditional froth flotation and oil agglomeration have been studied. Liu et al. (2002) developed the oily bubble flotation method, a process in which air bubbles are coated with thin oil films. By coating bubbles with an oil, flotation performance was improved as a result of the higher contact angle of the oil/particle surface. The stronger collecting power of the oil coated bubbles, over traditional air bubbles, resulted in lower processing times, however significant time was still required to achieve recoveries >95% (Wallwork et al., 2003). Gupta et al. (2016) have more recently developed the Hydrophobic-Hydrophilic Separation (HHS) process. Hydrophobic particles, such as coal, are transferred from a water phase to a hydrophobic liquid phase, generally a short chain alkane or oil. Coal agglomerates are formed in excess oil and then subsequently destabilized, such that entrapped water is released. The oil is then vaporized, condensed and recycled. Whilst being an efficient beneficiation process, multiple stages and significant energy input are required.

In other recent work, van Netten and co-workers have developed a novel hydrophobic binder for use in selective agglomeration. Originally developed with the aim of reducing the oil required in the selective agglomeration process, the hydrophobic binder has been shown to achieve rapid recovery across the full fine particle size range (van Netten et al., 2013, 2014, 2015, 2016). A high internal-phase (HIP) water in oil emulsion is used as the binder. This emulsion is characterized by a high internal phase volume fraction, i.e., >0.74. This is achieved by tightly packing water droplets within thin hydrophobic oil films. The tight packing, achieved through deformation of the drops, leads to internal phase volume fractions as high as 99% (Adamson, 1990).

Early work using the HIP water in oil emulsion binder showed a 2–3-fold (van Netten et al., 2013), followed by a 7-fold (van Netten et al., 2014) reduction in organic liquid requirements compared to conventional oil agglomeration in the recovery of coal particles. The most recent development however, resulted in a 10-fold reduction (van Netten et al., 2016). The hydrophobic emulsion binder consisted of a 95% dispersed aqueous phase and a 5% continuous organic phase. The aqueous phase consisted of a 3 wt% NaCl solution. The continuous organic phase contained equal mass portions of kerosene and emulsifier. The agglomeration process was remarkably complete within seconds, whilst traditional oil agglomeration needed residence times in the order of minutes.

To develop a clearer understanding of the hydrophobic emulsion binder functionality, further work was conducted using silica samples covering different particle size distributions (van Netten et al., 2017). A collector, CTAB, was added to the feed suspension to render the particles hydrophobic. The hydrophobic binder was then applied to the conditioned silica suspensions, achieving complete recovery of the particles in the form of agglomerates, using a mechanical screen to capture the solids. Silica particles ranging in size from 0.5 μm to >100 μm were recovered. The hydrophobic binder achieved a 16-fold reduction in oil consumption over conventional oil agglomeration for this material.

**Figure 1:**
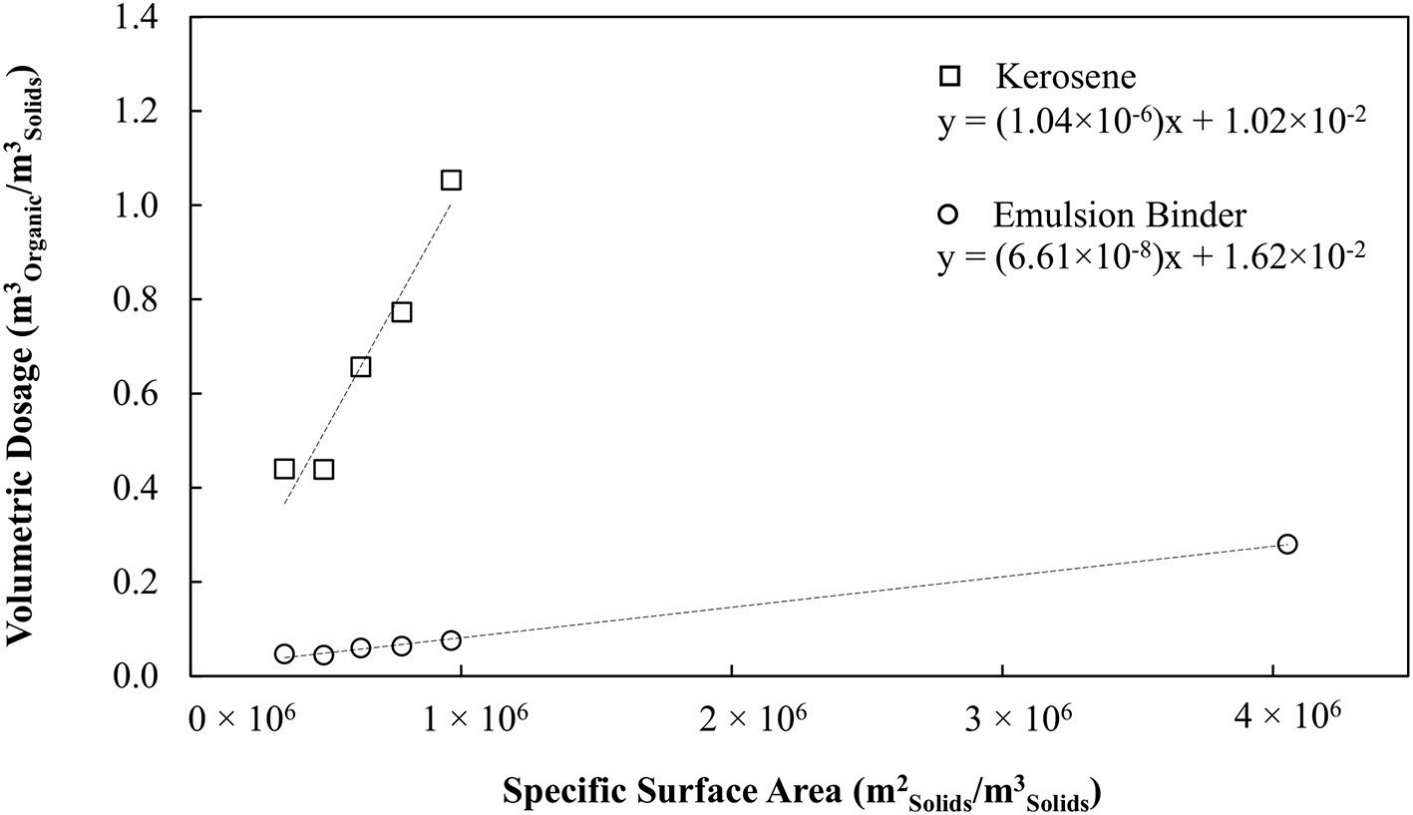
Organic liquid dosage as a function of specific surface area of solid particles. Adapted with permission from van Netten et al. (2017). Copyright 2017 American Chemical Society.

Figure 1 shows the oil consumption required to agglomerate the silica as a function of the specific surface area of the silica, for conventional oil agglomeration and for the hydrophobic binder. Figure 1 provides a measure of the effective film thickness of the oil over the particles via the slope. The effective film thickness with respect to the silica particle surface is 1,040 and 66.1 nm for the conventional and hydrophobic emulsion binders respectively, representing a 16-fold reduction in oil requirements. The water drops within the binder had a Sauter mean diameter of 2.3 microns, hence the average oil thickness with respect to the drops was 29 nm, corresponding to 58 microns between opposing water drops.

The hydrophobic emulsion binder provides the ideal delivery system for efficiently presenting the hydrophobic oil interface to the hydrophobic particles. In the form of a concentrated water in oil emulsion, the oil is present as thin films of order 50 nm, while in conventional oil agglomeration the mixing might only disperse the oil to form drops of order 1,000 nm, delivering a completely new system in which oil can coat and agglomerate hydrophobic particles. Thus, for the same volume of oil, the thin films wet a much larger surface area of particles, resulting in the decreased oil requirements and subsequent cost.

In the same study, van Netten et al. (2017) discovered that when the hydrophobic binder was subjected to intense mixing in water, and hence fragmented into smaller portions, water permeated into the binder fragments. Driven by the osmotic pressure gradient between the suspending water and the aqueous phase within the binder, which contained 3 wt % NaCl, water permeates across the thin oil films surrounding the dispersed drops within the binder. The viscous hydrodynamic resistance between the hydrophobic particles impinging on the binder surface effectively disappears due to the permeability of the hydrophobic interface. Thus, complete recovery of the hydrophobic particles across the full size range is observed within a few seconds.

Water transport through permeable films between an internal aqueous phase of a W/O emulsion and an external aqueous phase, i.e., a water-in-oil-in-water double emulsion, has previously been observed in the literature, with double emulsions used for drug release (Fukushima et al., 1983), cosmetics (Laugel et al., 1996), food (Garti, 1997), and agricultural fertilizers (Sela et al., 1994). However, in these applications it was desirable for the water (and water soluble material) to be transported from the internal aqueous phase to the external phase, i.e., the opposite direction to that which occurs in the agglomeration process using the hydrophobic binder (van Netten et al., 2017). Moreover, it appears that the work by van Netten et al. (2017) was the first to use the transport of water across a permeable hydrophobic interface for the simultaneous recovery of fine hydrophobic particles from suspension.

This study continues on from the previous work by van Netten and co-workers to examine the application of the hydrophobic binder to the recovery of magnetite from an aqueous suspension. This work represents the first successful attempt to recover a real mineral ore using the hydrophobic binder. Magnetite is naturally hydrophilic and thus requires a collector to render the surface hydrophobic. Typical aqueous collectors in magnetite flotation include fatty acids, hydroxamates and sulfates (Kulkarni and, 1980; Ma, 2012). Given that sorbitan monooleate (SMO) exists within the oil film as an emulsifier, the binder has the potential to simultaneously act as a collector for the magnetite. This means that hydrophilic particles of magnetite could literally attach to the hydrophobic binder due to the presence of the SMO at the interface. This mechanism is contrary to the so-called “hydrophobic interaction” acting to produce adhesion. Hence it is of interest to test this possible mechanism.

Impingement of the hydrophilic particles should damage the thin films while the hydrophobic particles should aid in the stability of the films. Ikem et al. (2008) have previously reported on the stabilization of HIP water in oil emulsions with silica particles hydrophobised by the adsorption of oleic acid, preparing stable emulsions with internal aqueous volume fractions of up to 95%. Moreover, Finkle et al. (1923) determined the relationship between a particle's hydrophobicity and its ability to stabilize either a water in oil (W/O) or oil in water (O/W) emulsion, with hydrophobic particles found to stabilize W/O emulsions. These findings are important here because it is of interest to maximize the recovery of hydrophobic particles while minimizing the extent to which the emulsion is degraded. Emulsion degradation is undesirable as it increases the effective organic liquid dosage required to achieve agglomeration.

The focus of the study is on how best to achieve full magnetite recovery, and to determine the minimum level of reagent and binder consumption. Ideally the magnetite can be recovered directly using the binder, with the SMO contained within the emulsion acting as a collector. This result will then be compared to the previous study, in which hydrophobic silica powder is agglomerated, to determine whether the binder addition is only dependent on the surface area of the particles. This will allow a model to be developed to determine the optimum binder requirements based on the specific surface area of the particles.

## Materials and methods

### Binder preparation

The emulsion was prepared as previously described by van van Netten et al. (2017), consisting of a dispersed aqueous phase volume fraction of 95% and a continuous organic phase volume fraction of 5%. First, equal portions of kerosene (Recochem Inc.,) and industrial grade emulsifier (Vic Chem) were combined in a stainless steel mixing bowl. The industrial grade emulsifier comprised of 60 wt% sorbitan monooleate and 40 wt% glycerol monooleate. The aqueous phase, 3 wt% sodium chloride (Cerebos Ltd.,) solution, was then incrementally added to the kerosene and emulsifier solution under constant mixing using a Russell Hobbs Hand Mixer (RHMX1). The aqueous sodium chloride solution was added in increments less than that of the original organic phase volume to insure phase inversion of the emulsion did not occur. As the binder volume increased, so did the addition of the aqueous phase. A viscous, opaque, high internal phase emulsion then formed.

### Agglomeration experiments

Agglomeration experiments were undertaken using a Waring high-speed blender (model LB20EG) operated at 22,000 rpm. Magnetite particles (Sibelco) with a Sauter mean diameter of 11.4 μm were added to 500 mL of tap water to produce an aqueous suspension. The aqueous suspension contained 60 g of solid particles in all experiments. The emulsion binder was then added in a single dose, with mixing occurring for 7 s. Since the emulsifier contained in the binder, sorbitan monooleate, acts as a collector for magnetite particles, no particle conditioning was used. At the conclusion of mixing, the contents of the blender were then poured over a 150 μm screen. The material retained on the screen was classified as the agglomerated product, whilst the material passing through was classified as the non-agglomerated reject. Both product and reject samples were dried in an oven (set at 110°C) and then weighed. The recovery value of an experiment was defined as the dry mass product as a percentage of the total dry feed. The binder dosage was progressively increased in order to determine the required organic liquid dosage to produce a fully agglomerated product.

### Emulsion degradation

To investigate emulsion degradation, the conductivity of the suspending liquid was measured using a EUTECH conductivity meter (PCTesr 35). Intense mixing leads to gradual degradation of the binder due to the stresses applied to the thin oil films. Progressively, salt contained within the internal aqueous droplets is released into the outer suspending liquid. By measuring the conductivity, the extent of the salt released can be measured and used to quantify the degradation. The maximum theoretical mass of salt in the emulsion was added to 500 mL of water and the conductivity measured. This was done to normalize the conductivity measurements. Systems that contained no particles (i.e., only the emulsion binder) and hydrophobic particles that are believed to stabilize the binder were used. Mixing occurred between 1 and 30 s in the Waring variable speed blender, operated at 22,000 rpm. The contents of the blender were then filtered so that no binder fragments or solids passed through into the filtrate used for the conductivity measurements.

## Results and discussion

**Figure 2:**
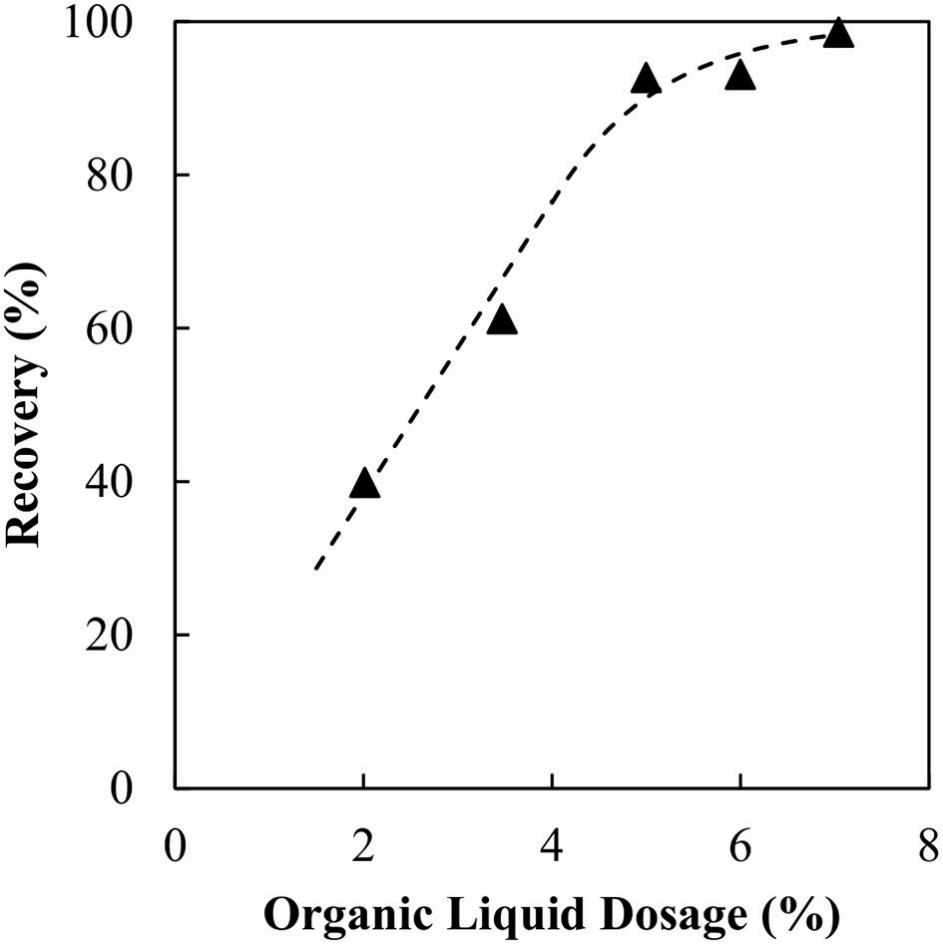
Magnetite recovery as a function of organic liquid dosage.

The recovery of magnetite as a function of the organic liquid dosage is shown in Figure 2. It is assumed in this case that the sorbitan monooleate within the binder acts as a collector, thus no pre-conditioning of the particles with a surfactant was required. A simple curve fit has been added to “guide the eye.” The optimum organic liquid dosage was determined by fitting the values of (100% - recovery) to a simple exponential decay function. The dosage at 90% recovery was chosen as the optimum organic liquid dosage. For the magnetite particles the optimum dosage was determined to be 5.5 wt% organic liquid. This result appears to be remarkable, recovering hydrophilic particles using a hydrophobic binder. The Supplementary Material describes the method for determining the organic liquid dosage requirements in further detail.

It is evident that increasing the organic liquid dosage results in an increase in magnetite recovery as agglomerates. The trend is consistent with the findings in the previous coal and silica work. What is different here is that the magnetite is hydrophilic, hence there must be a mechanism that renders the particles hydrophobic. However, the organic liquid requirement needed to fully agglomerate the particles appears to be excessive. Comparing the results to our previous silica study, the dosage is 6 times higher for the same specific surface area.

It is now hypothesized that the magnetite particles, initially hydrophilic, act in two ways. Firstly, the hydrophilic magnetite particles collide with the emulsion, causing degradation of the binder, and hence release of the SMO. The released SMO then adsorbs at the magnetite surface, rendering the magnetite hydrophobic. Secondly, with the particles now hydrophobic, the excess emulsion then acts as the binder to form the agglomerated product. This mechanism helps to explain why the level of binder required was excessive.

**Figure 3:**
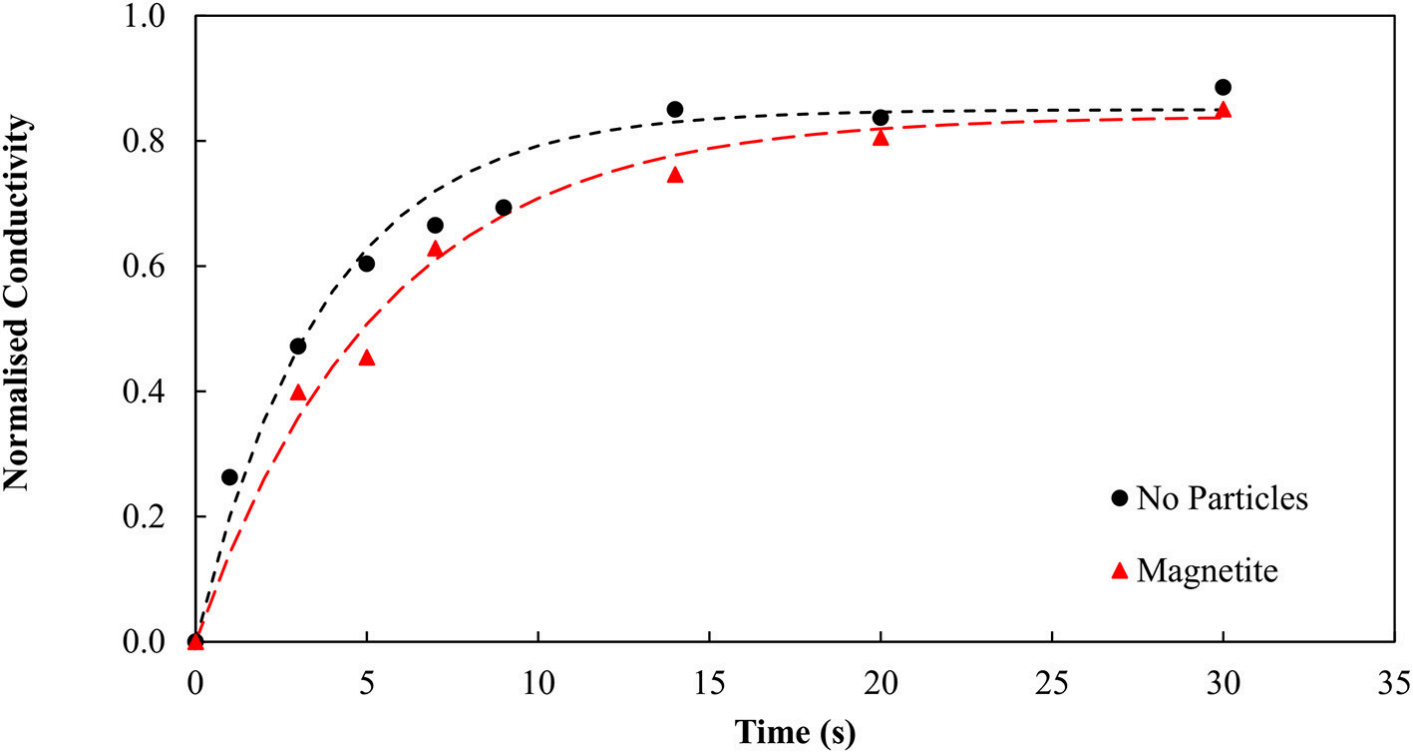
Emulsion degradation over time for systems containing no particles and magnetite particles.

Emulsion degradation experiments were therefore conducted in the presence of (a) no particles and (b) magnetite particles. It was initially believed that the impinging magnetite would stabilize the binder system. Figure 3 shows very similar trends for systems containing both particles and no particles. This result is contrary to the initial hypothesis that the magnetite particles should become hydrophobic, and in turn stabilize the system. Clearly the binder appears to degrade over time when there are no particles present, and when the magnetite is present.

**Figure 4:**
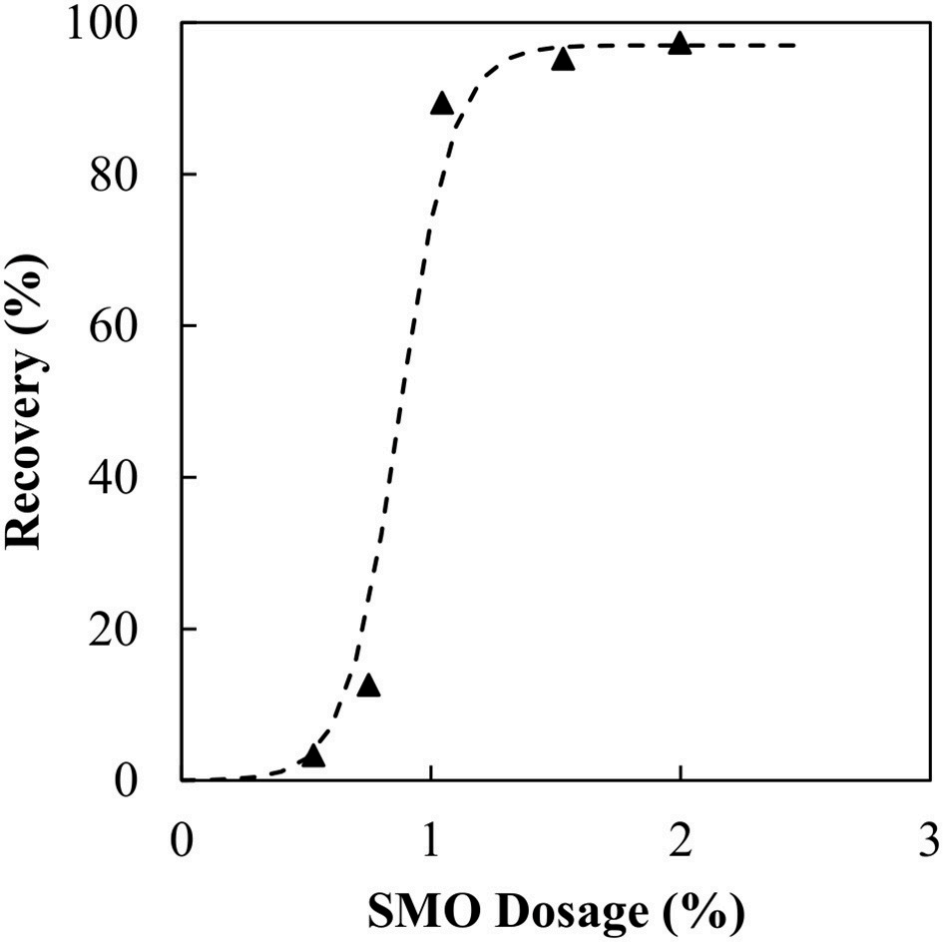
Magnetite recovery as a function of SMO collector concentration at predicted organic liquid dosage.

Thus, in subsequent experiments the magnetite particles were conditioned using SMO prior to the agglomeration with the binder. Here, the SMO acts as a collector, as in flotation, making the magnetite hydrophobic. In these new experiments, the particle conditioning occurred for 30 s at 22,000 rpm to disperse the SMO, followed by 30 min at 8,000 rpm. It is known that long conditioning periods are required to ensure the dispersed SMO adsorbs to the magnetite surface, making the particles hydrophobic (Kulkarni and, 1980), however, there was no optimisation here. The binder dosage was then reduced to the dosage predicted from previous work on a model silica system of the same specific surface area, i.e., ≈1 wt% organic liquid. In all experiments, the binder dosage remained constant. The SMO concentration in the magnetite solution was progressively increased from 0.5 wt% through to 2 wt% to find the optimum operating collector dosage. The influence of the SMO collector concentration can be seen in Figure 4.

**Figure 5:**
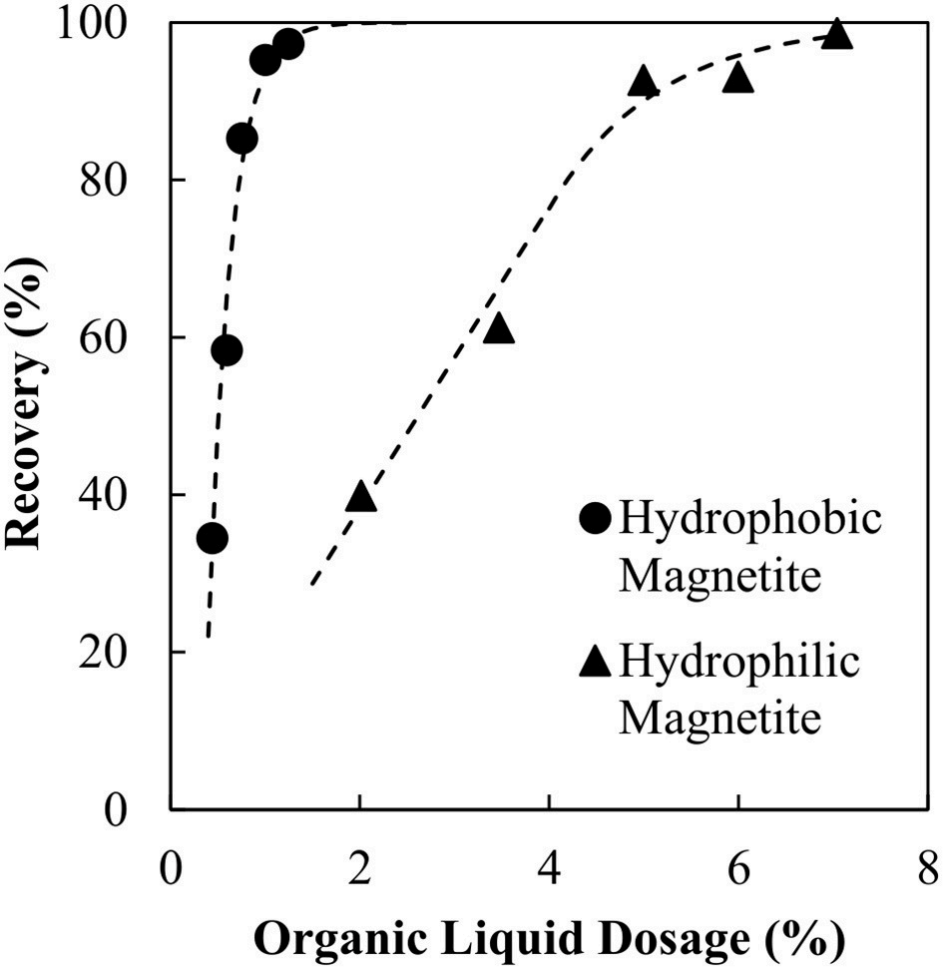
Reduction in organic liquid requirement for conditioned magnetite particles.

As the SMO dosage increases, so too does the recovery of magnetite. Initially between 0.5 and 0.75 wt% the increase in recovery is quite small before a sudden increase at 1 wt%. Initially there is insufficient SMO to render all of the magnetite particles hydrophobic. Therefore, with some hydrophilic particles still present, the hydrophobic particles deliver improved binder stability while the hydrophilic particles contribute to the binder degradation. Beyond 1 wt% all particles are hydrophobic, resulting in almost full recovery. The agglomeration experiments were then repeated for particles conditioned with 1.5 wt% SMO in the manner described above. Figure 5 presents the results from those experiments.

**Figure 6:**
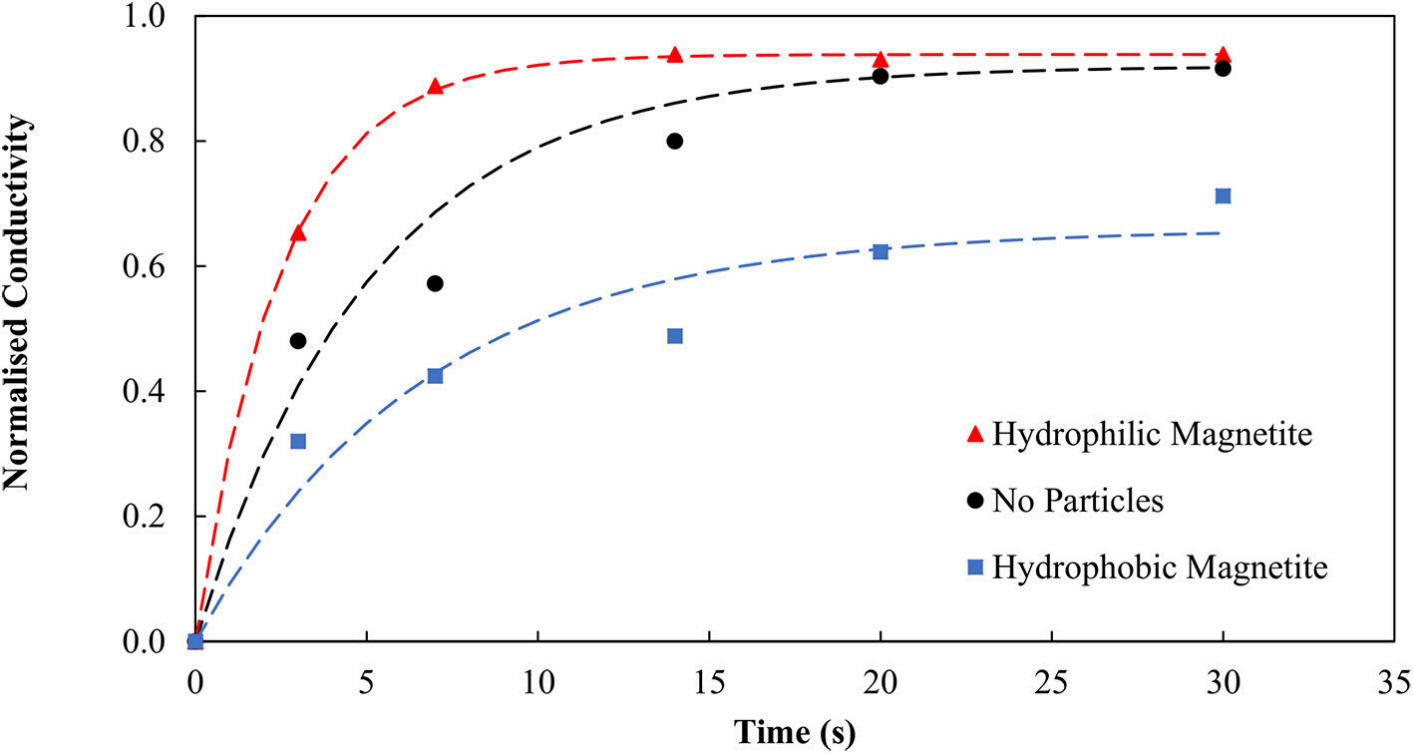
Emulsion degradation over time for systems containing no particles, magnetite particles and conditioned magnetite particles. Here the magnetite particles produced the most degradation. Previously, in Figure 3, the binder was present in excess hence the magnetite had less impact.

It is evident the organic liquid requirements are significantly reduced when the SMO is introduced directly as a collector and allowed to condition the particles prior to the agglomeration. By pre-dispersing the SMO as a collector, the binder functionality is preserved, resulting in the lower organic liquid dosage requirement. Emulsion degradation tests were conducted using the conditioned and thus hydrophobic magnetite particles and repeated for both no particles and the unconditioned, hydrophilic particles using the new optimum binder dosage of 0.98 wt% organic liquid to confirm the role of hydrophobic and hydrophilic particles in stabilizing and degrading the binder, respectively. Figure 6 provides evidence that the hydrophobic magnetite particles stabilize the binder, producing much lower conductivity over time, whilst the unconditioned and effectively hydrophilic magnetite particles produce much higher conductivity over time.

**Figure 7:**
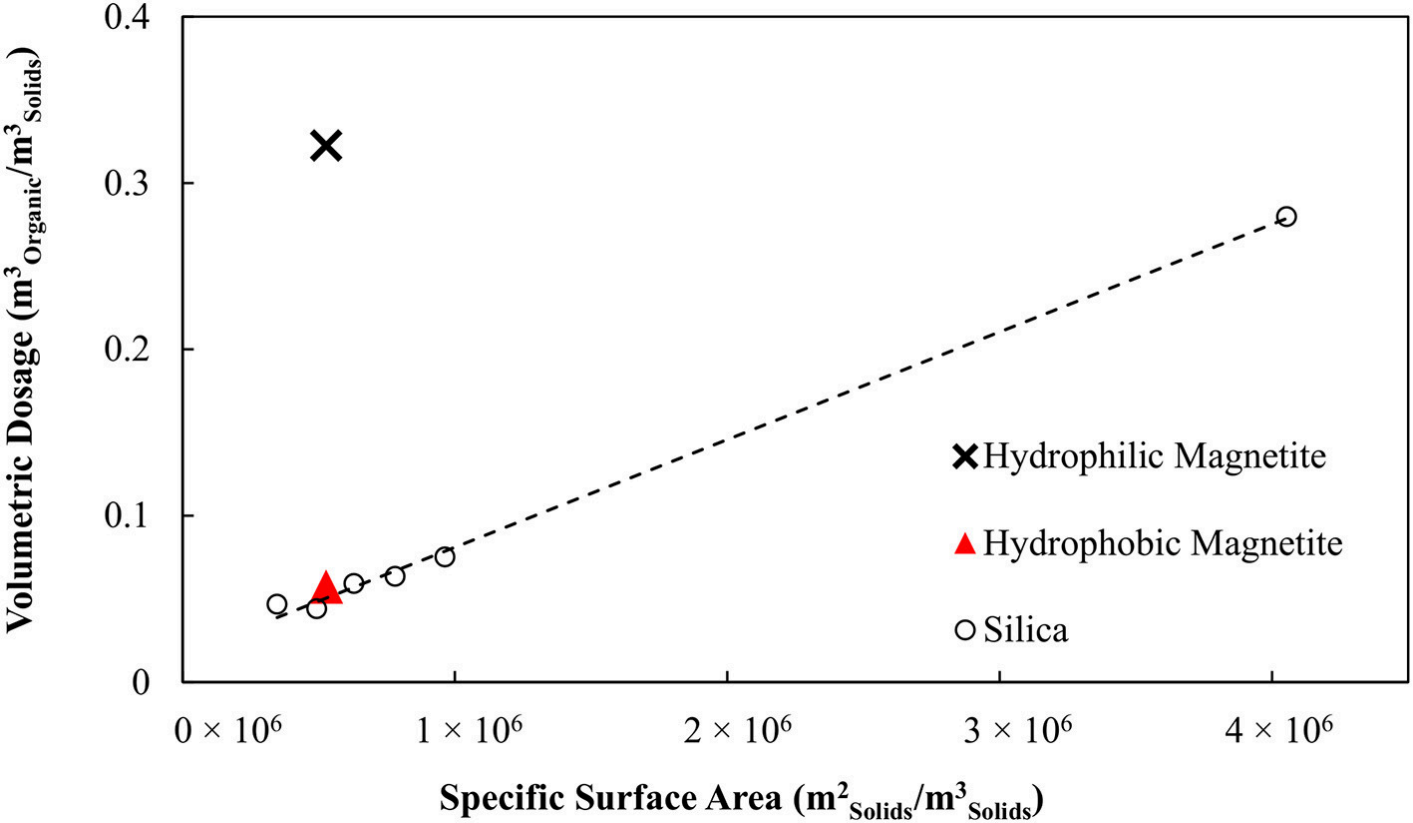
Comparison of organic liquid requirements for magnetite, magnetite conditioned with SMO and silica powders.

The organic liquid requirements of the binder as a function of the specific surface area of the hydrophobic particles is shown in Figure 7 together with the earlier result obtained for no collector. These data are also compared with the results obtained in the previous work by van Netten et al. (2017) for silica. The organic liquid requirement of the binder is excessive when no collector stage is used, 6-fold higher than for silica. However, when the magnetite is made hydrophobic in advance of the agglomeration, the organic liquid dose is almost the same as for the silica.

These findings support clear conclusions concerning the application of the binder to the recovery and concentration of magnetite. Firstly, the collector cannot be introduced via the binder. Rather, the collector must be added to the suspension prior to the agglomeration to form hydrophobic particles. Moreover, these results indicate that the binder dosage required to achieve agglomeration can be predicted for particles of any known specific hydrophobic surface area using the model derived from the silica data. This information can also assist in the determination of the correct collector dosage for new materials to which the agglomeration process is applied.

## Conclusions

In this work, an aqueous suspension of magnetite particles with a Sauter mean diameter of 11.4 μm was agglomerated using a high internal-phase water in oil emulsion binder. Previous studies in which both coal (van Netten et al., 2013, 2015, 2016) and hydrophobic silica (van Netten et al., 2017) were agglomerated with the novel binder have shown a number of advantages over flotation and conventional oil agglomeration. These include ultrafast agglomeration, reduced oil requirements compared to conventional oil agglomeration and the ability to recover a wide range of particle sizes, including the recovery of material as fine as 500 nm. This study aimed to apply our previously found knowledge from the silica model to a new feed source with industrial applications.

Firstly, knowing that SMO can act as a magnetite collector, a study to determine whether the emulsion could double as a collector and binder was undertaken. Remarkably the particles were agglomerated with no conditioning, however the binder dosages were excessive and higher than expected. It was found that there was a 6-fold increase in binder requirements compared to a silica powder of the same specific surface area. Further experiments, which examined emulsion degradation, showed that the magnetite did not stabilize the binder. The introduction of SMO as a collector was found to significantly reduce the organic liquid requirements needed for agglomeration. By conditioning the particles the binder requirements were found to be equal to those of a silica powder with the same surface area. Re-examining binder stability in the presence of the conditioned, now hydrophobic, particles showed the particles stabilized the binder.

It was also shown that the organic liquid dosage required to agglomerate the pre-conditioned magnetite particles matched that predicted by work on a model silica system. It was therefore concluded that it may be possible to use the model derived from the silica system to predict the binder requirements of any hydrophobic material, given the specific hydrophobic surface area is known. The finding showing hydrophilic particles degrade the binder, whilst hydrophobic particles provide stability, is important as this has implications for real systems that contain both hydrophobic and hydrophilic particles.

## Author contributions

All authors listed have made a substantial, direct and intellectual contribution to the work, and approved it for publication.

### Conflict of interest statement

The authors declare that the research was conducted in the absence of any commercial or financial relationships that could be construed as a potential conflict of interest.
