# Supplementary material for: Ultrafine Particle Recovery Using Thin Permeable Films
Source: Front Chem. 2018 Jun 19;6:220. doi: 10.3389/fchem.2018.00220 (PMC6018405; doi:10.3389/fchem.2018.00220)
Supplement: Supplementary file 1 [file Data_Sheet_1.DOCX]

Supplementary Material

Ultrafine particle recovery using thin permeable films

Daniel J. Borrow*, Kim van Netten, Kevin P. Galvin

*** Correspondence:** Daniel Borrow: daniel.borrow@uon.edu.au

# Supplementary Data

## Establishing the Organic Liquid Dosage Required to Achieve Agglomeration

In order to correlate the organic liquid dosage requirements for the magnetite particles with the specific surface area of the particles, a standardised method for determining the organic liquid dosage requirement from the recovery-dosage curves was developed. The method fitted the organic liquid dosage and (100 – %recovery) data to a simple exponential decay function. Supplementary Figure 1, presents the complete set of fitted curves for the unconditioned and SMO conditioned magnetite, respectively.


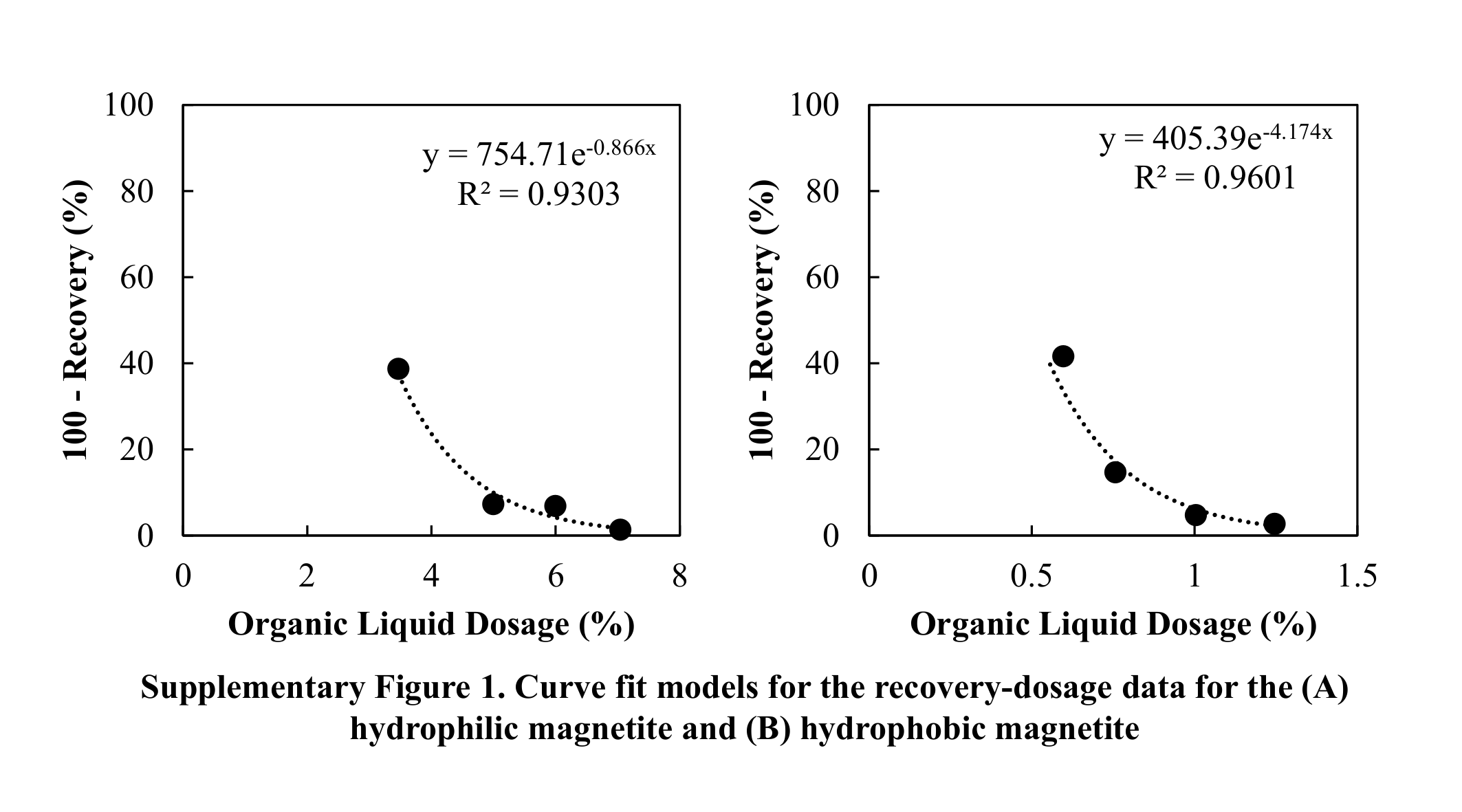
To establish the representative dosage for the particles using the binder, the dosage at 90% recovery was calculated using the fitted function for each data set. A recovery of 90% was selected as the basis for comparison as it sits in a very distinct part of the curve, immediately after the knee and prior to the plateau towards 100%. To serve as an example, the dosage (d) at this point was calculated for the unconditioned particles,

(100 – Recovery) = 754.71e^-0.866d^

(100 – 90) = 754.71e^-0.866d^

Thus, d = 4.99 wt% organic liquid

However, as this dosage was relative to the mass of feed material, the dosage had to be normalised to the mass of material recovered in the product. This normalisation was carried out to provide a more accurate representation of the binder-solid ratio required in the formation of agglomerates. The final organic liquid dosage for the unconditioned particles was therefore 4.99/0.9= 5.54 wt% organic liquid when using the emulsion binder.
