# Supplementary figures and images for: Ultrafine Particle Recovery Using Thin Permeable Films
Source: Front Chem. 2018 Jun 19;6:220. doi: 10.3389/fchem.2018.00220 (PMC6018405; doi:10.3389/fchem.2018.00220)

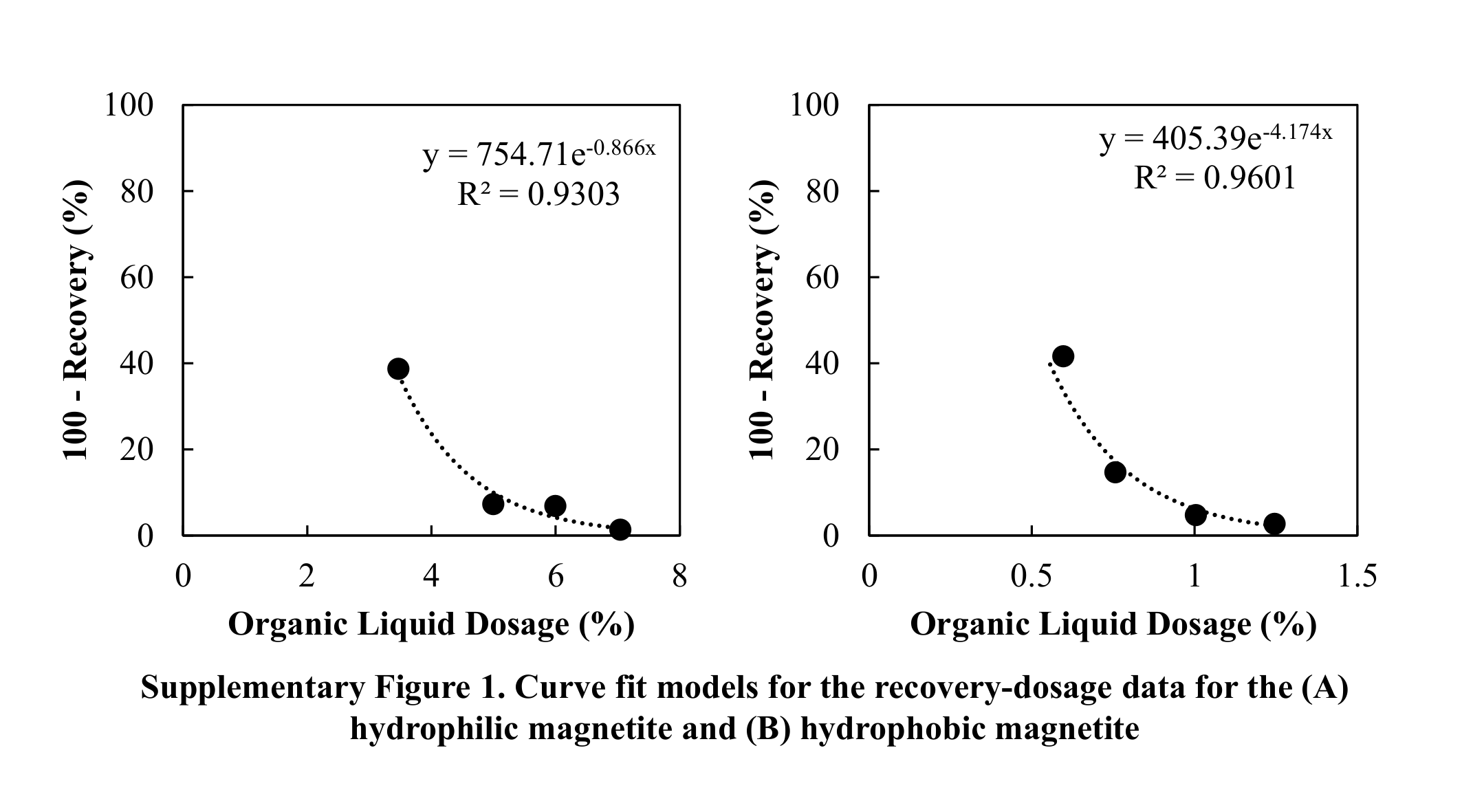

Supplement: Supplementary file 2 [file Image_1.TIF]
